# Editorial: Closure and Reopening of Schools and Universities During the COVID-19 Pandemic: Prevention and Control Measures, Support Strategies for Vulnerable Students and Psychosocial Needs

**DOI:** 10.3389/fpsyg.2021.769622

**Published:** 2021-10-12

**Authors:** Elena Commodari, Valentina Lucia La Rosa, Maria Anna Coniglio, Daniela Conti

**Affiliations:** ^1^Department of Educational Sciences, University of Catania, Catania, Italy; ^2^Department of Medical and Surgical Sciences and Advanced Technologies “G.F. Ingrassia”, University of Catania, Catania, Italy; ^3^College of Business, Technology and Engineering, Sheffield Hallam University, Sheffield, United Kingdom

**Keywords:** COVID-19, school, university, prevention, health risk perception, vulnerability, psychosocial needs, distance learning

The pandemic event and the consequent lockdown in many countries of the world have raised new issues and, at the same time, stimulated the debate on essential aspects of educational psychology that need to be declined with a specific reference to COVID-19. According to the United Nations Educational, Scientific and Cultural Organization (UNESCO), school and university closures impacted over 90% of the world's student population. In many countries, governments have activated measures to maintain continuity of learning using different forms of distance learning. However, thanks to the introduction of vaccinations to contain the pandemic, schools and universities are starting to resume their activities, adopting varying degrees of strict measures to prevent and control the infection.

In this context, it is necessary to create a reflection on the reopening of schools and universities and on the most appropriate measures to be adopted to manage reopenings effectively, as well as on interventions to support situations of greater vulnerability. Therefore, it is important to focus on the aspects of prevention and promotion of good risk management behaviors, on support measures for the most vulnerable students such as those with special educational needs and disabilities, and on the psychosocial needs that a stressful event such as a pandemic can result in for students, families, and teachers.

The strong impact of the COVID-19 pandemic on the psychological well-being of university students worldwide was confirmed by several studies. In this regard, the study by Awoke et al., conducted on undergraduate health science students of Jimma University (Ethiopia), underlined that over one-third of the participants reported high perceived stress. Furthermore, Swiss university students also reported high levels of anxiety and depression, especially during the early stages of the pandemic, as shown in the study by Amendola et al. In particular, the authors found that older age, female gender, non-Swiss nationality, loneliness, and participants' concern about their health positively predicted anxiety. In contrast, resilience and social support negatively predicted anxiety.

The same finding is also confirmed by Wu et al.'s study of 14,769 Chinese university students. Specifically, the authors reported that the increases in anxiety and depression from pre-pandemic levels were associated with students' gender and the severity of the pandemic in the province where they resided. Finally, De Pasquale et al.'s studies emphasized that the pandemic's experience can be a risk factor for the emergence of problematic behaviors among Italian university students. In particular, the first study reported that anxiety significantly correlated with bulimic behavior, while depression correlated with impulsivity and binge eating behaviors (De Pasquale et al.). The second study confirmed that Italian university students also showed moderate trait and state anxiety, as well as moderate perceived vulnerability to disease (De Pasquale et al.). However, fear of COVID-19 and trait anxiety did not seem to predict the risk of smartphone addiction.

The study by Carpinelli et al. added an important contribution to the Research Topic by focusing on the pandemic experiences of Italian university students with disabilities and specific learning disabilities. The authors reported high levels of satisfaction with emergency remote teaching during the lockdown phase among these students. Furthermore, only 22% of them indicated that they were dissatisfied with the teaching method used.

These results confirm that the psychological well-being of university students should be carefully considered. Therefore, it is necessary to provide adequate crisis-oriented psychological services to support this specific population in addressing the uncertainty associated with the pandemic. In this regard, the brief research report by Rusch et al. is very interesting because it reports the experience of the University of Michigan that activated a school mental health implementation program (TRAILS) designed to improve youth access to evidence-based mental health services. In particular, this report examined the needs of school mental health professionals of the University of Michigan during the early months of the COVID-19 pandemic and how those needs contributed to improving programming and resources provided by the TRAILS program.

In addition, other studies have contributed to investigating some relevant variables associated with the psychological impact of the pandemic and the experience of distance learning among university students. First of all, learning strategies influence students' online learning satisfaction through academic emotions during the COVID-19 pandemic, as reported in the study by Wu et al.. Staller et al. underlined that morning-oriented, conscientious, and open students with low neuroticism seem to better cope with the changed learning situation due to vitality, self-efficacy, and partly their self-determined motivation. Furthermore, according to Zhang et al., adaptability and student engagement are significantly positively correlated with positive academic emotions and negatively correlated with negative ones. As underlined by Wang et al., digital competence is another crucial variable to consider because it indirectly affected academic burnout through its effect on cognitive load and showed a great positive influence on student engagement. The study by Zeng et al. explored the impact of post-traumatic growth on college students' creativity during the COVID-19 pandemic. In this regard, the authors reported that post-traumatic growth affected creativity directly and indirectly through self-efficacy. This association was stronger when the incidence of deliberate rumination was low. According to these findings, the adverse effects of the COVID-19 pandemic can be alleviated through positive psychological interventions on university students to promote these dimensions.

Finally, the interesting contribution by Zhang and Huang focuses on a more specific aspect, namely the impact of the entrepreneurial environment on entrepreneurial self-efficacy and intentions of college students in the post-pandemic era. The authors reported that the factors influencing the entrepreneurial choice of college students included gender, entrepreneurial family history, major, and educational background. Furthermore, entrepreneurial self-efficacy can significantly mediate the impact caused by the post-pandemic entrepreneurial environment on entrepreneurial intentions.

In conclusion, the educational impact of an extraordinary and unexpected event such as the COVID-19 pandemic needs to be deeply investigated. Future studies will have to explore this topic further, especially in light of the reopening of schools and universities, to adapt the organization of teaching and academic life according to students' new needs and experiences.

## Author Contributions

EC and VLLR were guest associate editors of the Research Topic and wrote the paper text. MC and DC were guest associate editors of the Research Topic and edited the text. All authors contributed to the article and approved the submitted version.

## Conflict of Interest

The authors declare that the research was conducted in the absence of any commercial or financial relationships that could be construed as a potential conflict of interest.

## Publisher's Note

All claims expressed in this article are solely those of the authors and do not necessarily represent those of their affiliated organizations, or those of the publisher, the editors and the reviewers. Any product that may be evaluated in this article, or claim that may be made by its manufacturer, is not guaranteed or endorsed by the publisher.

